# Primary Cilium Depletion Typifies Cutaneous Melanoma In Situ and Malignant Melanoma

**DOI:** 10.1371/journal.pone.0027410

**Published:** 2011-11-11

**Authors:** Jinah Kim, Salma Dabiri, E. Scott Seeley

**Affiliations:** 1 Department of Pathology, Stanford University Medical Center, Stanford, California, United States of America; 2 Department of Dermatology, Stanford University Medical Center, Stanford, California, United States of America; United States of America

## Abstract

Cutaneous melanoma is a lethal malignancy that arises spontaneously or via in situ precursor neoplasms. While melanoma in situ and locally invasive malignant melanoma can be cured surgically, these lesions can sometimes be difficult to distinguish from melanocytic nevi. Thus, the identification of histolopathologic or molecular features that distinguish these biologically distinct lesions would represent an important advance. To this end, we determined the abundance of melanocytic primary cilia in a series of 62 cases composed of typical cutaneous melanocytic nevi, melanoma in situ, invasive melanoma, and metastatic melanoma. Primary cilia are sensory organelles that modulate developmental and adaptive signaling and notably, are substantially depleted from the neoplastic epithelium of pancreatic carcinoma at a stage equivalent to melanoma in situ. In this series, we find that while nearly all melanocytes in 22 melanocytic nevi possessed a primary cilium, a near-complete loss of this organelle was observed in 16 cases of melanoma in situ, in 16 unequivocal primary invasive melanomas, and in 8 metastatic tumors, each associated with a cutaneous primary lesion. These findings suggest that the primary cilium may be used to segregate cutaneous invasive melanoma and melanoma in situ from melanocytic nevi. Moreover, they place the loss of an organelle known to regulate oncogenic signaling at an early stage of melanoma development.

## Introduction

Melanoma accounts for approximately 4% of skin neoplasms but 80-90% of skin cancer deaths. While the prognosis of disseminated melanoma is bleak, prospects for the cure of locally invasive and in situ melanoma by surgical approaches are excellent. Thus, improved recognition and understanding of the key biological steps that initiate melanomagenesis are critical. However, early stage melanomas can at times be challenging to distinguish morphologically from melanocytic nevi, which are also known aspigmented moles. Accordingly, diagnostic discordance rates as high as 25% have been reported when case series have been reviewed by multiple pathologists [1], [2]. Moreover, because between 1 and 2 million biopsies are performed each year to evaluate for melanoma and there exist strong medicolegal incentives to overdiagnose this malignancy, a substantive cost-burden is placed on the heath care system and many patients are subjected to unnecessary treatment, surgical procedures, and disease monitoring. Conversely, missed melanoma diagnoses result in preventable morbidity and mortality. Thus, the continued development of ancillary testing modalities able to identify early events in melanomagenesis is a high priority.

Primary cilia are ubiquitous sensory organelles that coordinate adaptive and developmental signaling by recruiting, organizing, and mobilizing diverse classes of signaling molecules in response to extracellular ligands [3]. Indeed, the growing list of receptors known to localize to primary cilia includes those of the Hedgehog, Wnt, somatostatin, serotonin, PDGF, and EGF signaling pathways [4]. Structurally, the primary cilium is composed of a 9+0 microtubular axoneme ensheathed by a membrane that is contiguous with, but biochemically distinct from, the plasma membrane [5]. The microtubule doublets of the ciliary axoneme are assembled atop and continuous with two of the three triplet microtubules of the maternal centriole. Thus, primary cilia are always found in association with a maternal centriole. Moreover, because ciliary tubulins undergo extensive post-translational modification, antibodies directed towards modified tubulins, including acetylated, polyglutamylated, and detyrosinated forms, can be used in conjunction with antibodies recognizing centriolar proteins to identify primary cilia with a high degree of specificity [6], [7].

Defects in primary cilium assembly and function are associated with profound developmental disorders including Bardet Biedl Syndrome, Alstrom Syndrome, and polycystic kidney disease [8]. However, relationships between this organelle, its associated transport machinery, and cancer are just beginning to be explored. In the first thorough study of the fate of this organelle during cancer development, we found that primary cilium assembly is actively suppressed by excessive Kras signaling in nearly all neoplastic epithelial cells during pancreatic ductal adenocarcinoma development, beginning with the appearance of its major in situ neoplasm [6]. However, more recent studies have shown variable frequencies of occurrence of primary cilia amongst the neoplastic cells of basal cell carcinoma, medulloblastoma, and renal carcinoma [9]-[11]. More historical accounts demonstrate that pituitary adenocarcinomas and ovarian Brenner tumors are ciliated and, indeed, there are examples in which cells appear to have acquired, rather than lost, the ability to ciliate following transformation [12]. Overall, the variation in the behavior of the primary cilium that follows neoplastic transformation may reflect variable requirements for the organelle in the oncogenic signaling pathways recruited by particular tumors and/or the particular cell type that has become transformed.

Interestingly however, pancreatic ductal adenocarcinoma and cutaneous melanoma have similar genetics, both exhibiting increased incidence in kindreds bearing heritable p16^Ink4a^ and other mutations. Moreover, melanoma and pancreatic ductal adenocarcinoma are frequently driven by activating mutations in components of the Ras signaling pathway [13]–[16] and we have shown that excessive Ras signaling can actively suppress cilium assembly in pancreatic ductal adenocarcinoma cells [6]. Therefore, because primary cilia frequently appear on normal cutaneous melanocytes [17], [18], we sought to determine whether a pattern of cilium loss analogous to that seen in pancreatic carcinoma development might permit the segregation of cutaneous melanoma and melanocytic nevi. In this study, we quantitated melanocytic primary cilia in a series of 62 cutaneous cases composed of typical melanocytic nevi, melanoma in situ, invasive melanoma, and metastatic melanoma of known cutaneous origin.

## Results

To identify differences in the ciliation status of melanocytes present in distinct melanocytic lesions, we collected 22 cases of melanocytic nevi, 16 cases of melanoma in situ, and 16 cases of invasive melanoma and subjected them to immunofluorescence microscopy. These specimens were costained with antibodies directed towards Sox10, to detect melanocytes [19], [20], acetylated or detyrosinated alpha tubulin, to detect the modified tubulins of the ciliary axoneme [21], [22], and gamma tubulin or CP110, to detect centrioles and basal bodies [23]. To assess the abundance of primary cilia, we considered any elongated acetylated or detyrosinated tubulin -containing structure in association with CP110 or a gamma tubulin -positive puncta to be a primary cilium. Because cytoplasmic microtubules can also contain modified tubulins [21], [22], this consideration ensured that primary cilia, and not cytoplasmic microtubules, were counted. Because tubulin acetylation is not obligate for axoneme assembly [24], it was also important to utilize multiple markers of the ciliary axoneme. Finally, because centriolar microtubules can also contain modified tubulins [25], we considered acetylated or detyrosinated tubulin puncta to represent centrioles and not primary cilia, though we appreciate that such structures may in fact represent extremely short ciliary axonemes. Nevertheless, this strategy was sufficient to identify unequivocal differences in the ciliation status of melanocytes present in the distinct types of lesions under study here.

**Figure 1:**
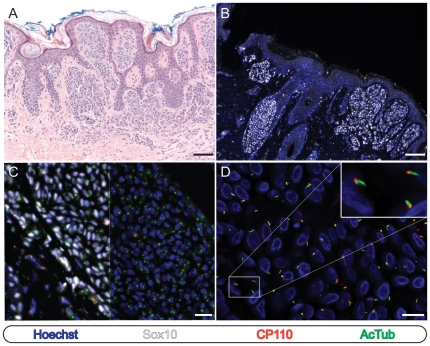
Primary cilia are retained by the melanocytes of typical compound and junctional cutaneous melanocytic nevi. Immunostaining as indicated. (A and B) Low-power images of cutaneous melanocytic nevi stained with hematoxylin and eosin (A) or for immunofluorescence microscopy (B). Scale bars: 100 µm. (C) Split image of nested melanocytes within a melanocytic nevus. Sox10 epifluorescence has been omitted from the right half of the image to enhance visualization of cilia and centriole staining. Scale bar: 50 µm. (D) High power image of nest shown in panel C. Scale bar: 25 µm.

When examined by immunofluorescence microscopy, we found that an average of 94% (range: 91% - 100%; SD: 3.06) of the Sox10 -positive melanocytes present in any of the 22 melanocytic nevi examined bore a primary cilium (figures 1 and S2). Identical results were obtained regardless of whether detyrosinated or acetylated tubulin antibodies were utilized (figure S1). We also identified primary cilia on non-melanocytic Sox10 -negative cells in regions adjacent to the melanocytic nevi in each specimen (not shown). In contrast, when dermal lymphocytes and other leukocyte populations were examined, detyrosinated and acetylated tubulin positive centrioles were observed but never a primary cilium, a finding consistent with the fact that leukocytes as a class have never been observed to ciliate (not shown). Together, these findings highlight the specificity of our staining and scoring system and demonstrate that benign melanocytes are ciliated.

**Figure 2:**
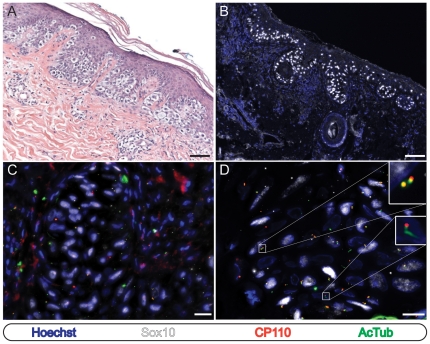
Melanocytes in melanoma in situ frequently fail to possess a primary cilium. Immunostaining as indicated. (A and B) Low-power images of cutaneous melanoma in situ stained with hematoxylin and eosin (A) or for immunofluorescence microscopy (B). Scale bars: 100 µm. (C) Melanoma in situ cells. Scale bar: µm. (D) High power image of melanoma. Upper inset: Sox10 positive melanoma in situ cell showing absence of ciliated centrioles. Lower inset: Sox10 negative cell with ciliated centriole.

**Figure 3:**
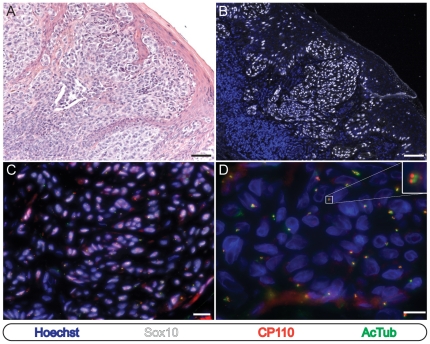
Melanocytes in invasive melanoma frequently fail to possess a primary cilium. Immunostaining as indicated. (A and B) Low-power images of invasive cutaneous melanoma stained with hematoxylin and eosin (A) or for immunofluorescence microscopy (B). Scale bars: 100 µm. (C) Invasive melanoma cells. Scale bar: 50 µm. (D) High power image of invasive melanoma. Upper inset: Invasive melanoma cell showing absence of ciliated centrioles.

We next examined a set of 32 cutaneous melanocytic proliferations comprised of 16 cases of cutaneous melanoma in situ and 16 primary invasive malignant melanomas. As seen in melanocytic nevi, we observed ciliated Sox10-negative cells (figure 2D, lower inset) and non-ciliated leukocytes (not shown) in all 16 cases, providing an internal positive control for each specimen. In contrast however, an average of 5% (range: 0%–9.5%; SD: 4.3) and 3% (range: 0%–9%; SD: 2.9) of the neoplastic Sox10 -positive melanoma in situ and invasive melanoma cells, respectively, were ciliated (figures 2, 3, and S2). Again, identical results were obtained regardless of whether detyrosinated or acetylated tubulin antibodies were utilized (figure S1). These findings demonstrate a uniform loss of the primary cilium amongst the neoplastic melanocytes of cutaneous melanoma in situ and malignant melanoma and suggest that primary cilium assembly is suppressed during the initiation of cutaneous melanoma.

**Figure 4:**
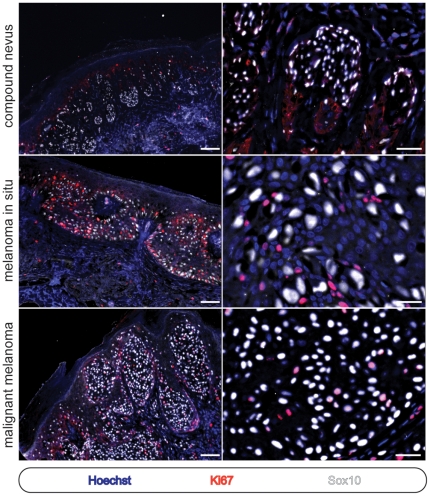
Cell cycle activity within melanocytic proliferations. See text for quantitation and further details. Immunostains and lesion types as indicated. Scale bars, left panels: 100 µm. Scale bars, right panels: 50 µm.

Primary cilia can be either present or absent during progression through interphase [4]. Thus, we were interested in learning whether the observed loss of melanocytic primary cilia might be due to ongoing cell cycle progression or instead, as an alternative consequence of neoplastic transformation. To this end, we performed Ki67 staining to compare the proliferative and ciliation indices of the neoplastic melanocytes present in each case. Ki67 is a protein that is present in the nucleus throughout the cell cycle, including mitosis, but is absent during G0. Thus, were the loss of the primary cilium to occur as a consequence of ongoing proliferation we would expect the proportion of non-ciliated cells to closely match the proportion of Ki67 -positive cells. In agreement with prior reports documenting the highly variable proliferation indices of melanocytic neoplasms [26]–[28], we found average proliferative indices of 0.4% (range: 0%–3.2%; SD: 0.89), 8.4% (range: 2.4%–22.2%; SD: 7.0), and 37.4% (range: 11.6%–43.4%; SD: 8.3) amongst our cases of melanocytic nevi, melanoma in situ, and invasive melanoma, respectively (figures 4 and S2). Therefore, and similar to that observed in pancreatic ductal adenocarcinoma, the vast majority of the melanoma in situ and invasive melanoma cells failed to ciliate, while as few as 2% were actively progressing through the cell cycle. Thus, insofar as Ki67-negative cells are not actively progressing through the cell cycle, active cell cycle progression is unlikely to primarily account for the loss of primary cilia observed in melanoma. However, it remains formally possible that recent cell cycle activity could somehow exert a residual negative influence on cilium assembly or maintenance in melanoma cells.

**Figure 5:**
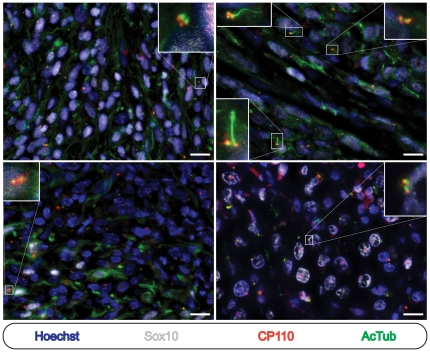
Metastatic melanoma cells of cutaneous origin frequently fail to possess a primary cilium. Immunostaining as indicated. Four separate lymph node metastases of distinct morphologies from 4 separate patients are shown. Insets show ciliated or unciliated centrioles of Sox10 positive metastatic melanoma cells. Scale bars: 50 µm.

Lastly, we were interested in learning whether the marked reduction in primary cilia seen in primary cutaneous melanocytic neoplasms might be seen in metastatic melanomas of known cutaneous origin. As such, we examined a series of involved lymph nodes from 8 patients with primary cutaneous invasive melanoma. Similar to the in situ and invasive cutaneous melanoma cases examined, we found that few metastatic melanocytes bore a cilium (figure 5). While we noted that primary cilia were slightly more apparent in metastatic lymph node deposits as compared to primary melanomas (figure 5), we did not encounter a metastatic deposit in which greater than 10% of the Sox10 positive cells were ciliated. Thus, primary cilia are substantially depleted from melanoma cells at all stages of cutaneous melanoma development and progression.

## Discussion

This study adds an additional chapter to the growing literature describing the fate and function of primary cilia in cancer development. In this study, we determined that primary cilia are strikingly depleted during the development of cutaneous melanoma. By costaining for centriolar proteins and modified tubulins of the ciliary axoneme we have found that, while primary cilia are assembled by nearly all melanocytes contained within cutaneous melanocytic nevi, they are lost from the vast majority of those associated with cutaneous melanoma in situ, invasive melanoma, and metastatic melanoma of dermal origin. Moreover, insofar as at least 90% of the neoplastic melanocytes present in any single lesion studied here were not ciliated while only 2%–43% were actively progressing through the cell cycle as determined by Ki67 immunostaining, ongoing cell cycle progression does not account for the loss of the primary cilium during melanomagenesis. However, it remains formally possible that recent cell cycle activity may somehow exert a residual negative influence on cilium assembly or maintenance in melanoma cells.

Our findings provide an interesting parallel between pancreatic adenocarcinoma development and melanoma with regard to the ciliation status of in situ and malignant cell types and demonstrate clear differences between cutaneous melanocytic nevi and melanoma. However, several important questions remain.

One, at which stage during melanocyte transformation is the primary cilium lost? The natural histories of cutaneous melanocytic nevi and melanoma in situ have proven difficult to characterize and moreover, represent an area of ongoing controversy. Because it remains possible that melanoma in situ undergo a transient ciliated phase, it will be important to examine a larger case series of cutaneous nevi and melanoma in situ in order to determine whether ciliated melanoma in situ or unciliated cutaneous nevi occur. Such studies would help determine whether assays of primary cilium abundance may be developed and ultimately applied to the early diagnosis of melanoma.

Two, while we have examined typical cutaneous melanocytic nevi and melanoma, the more diagnostically challenging melanocytic proliferations include those with with ambiguous histopathological features. These lesions are of particular interest with regard to their ciliation status because they exhibit unpredictable malignant potential. Therefore, we are currently working to determine whether quantitative or qualitative relationships exist between primary cilia, dysplasia, and clinical outcome in the setting of these lesions.

From a biological perspective, the implications of this study are less clear but certainly raise interesting questions regarding the initiation and progression of cutaneous melanoma. While biallelic deletion of genes required for the assembly and function of primary cilia dysregulates pathways important in cancer development [3] and can modify the tumorigenic potency of hedgehog pathway mutations [9], [10], the relationship between the primary cilium and the development of cancers not bearing hedgehog mutations is unclear. In this regard, it is interesting that pancreatic carcinoma and cutaneous melanoma thus far appear to exhibit analogous patterns of cilium depletion during their development. While pancreatic carcinoma and cutaneous melanoma are of highly divergent embryologic origin, both are most frequently associated with mutations inducing constitutive Ras pathway activation and both are associated with frequent loss of function mutations or epigenetic silencing of the p16 tumor suppressor. It is likely that comparative analyses of these embryologically divergent cancers may hasten the discovery of the mechanisms by which cilium assembly is suppressed during their development and the isolation of key oncogenic or tumor suppressive functions of primary cilia.

Overall, while additional studies will be necessary to firmly establish diagnostic and biological roles for primary cilia in melanomagenesis, this study demonstrates that primary cilia are depleted from cutaneous melanoma in situ, invasive melanoma, and metastatic cutaneous melanoma cells and places the widespread loss of an important regulator of oncogenic signaling at an early phase of melanoma development.

## Materials and Methods

### Clinical Samples

All clinical samples were procured according to a protocol approved by the Stanford University Research Compliance Office Human Subjects Research Compliance Panel (FWA 00000935) from the archives of the Stanford Department of Pathology. All patient information associated with this study was obtained in de-identified format and, as such, study-specific patient consent was not obtained. All cases carried a prior pathological diagnosis rendered by a board-certified dermatopathologist and were verified histologically by J.K. and S.D.

### Microscopy

7 µm-thick sections were cut, deparaffinized in xylene, rehydrated though graded ethanol dilutions, and subjected to hematoxylin and eosin staining according to standard procedures. Immunostaining was performed as described [6], with acetylated tubulin (Sigma), detyrosinated tubulin (Covance), gamma tubulin (Sigma), CP100 (gift from B.Dynlacht), Sox 10 (Santa Cruz Biotechnology), and Ki67 (AbCam) antibodies used at 1∶2000, 1∶500, 1∶200, 1∶200, 1∶200, and 1∶200, respectively. Alexafluor 488, 594, and 647 chicken anti -mouse, -rabbit, and -goat antibodies were used as secondary antibodies at a concentration of 1∶300 each. Slides were then mounted using PermaFluor mounting medium (Thermo) and stored at room temperature. Imaging was performed as described in “Results” using a Zeiss LSM META confocal and an Olympus BX series upright fluorescence microscope.

## Supporting Information

Figure S1 **Detyrosinated tubulin antibodies show the same pattern of cilium loss in neoplastic melanocytes as do acetylated tubulin antibodies.** Immunostaining and lesion types as shown. Scale bars: 20 µm.
(TIF)

Figure S2 **Proliferative indices do not fully account for the loss of the primary cilium amongst neoplastic melanocytes.** (L) Average ciliation indices of melanocytic proliferations expressed as the percent of lesional melanocytes that are ciliated. (R) Average Ki67 proliferation indices of melanocytic proliferations expressed as the percent of lesional melanocytes that exhibit nuclear Ki67 immunoreactivity. Error bars: Standard deviation of mean.
(JPG)
