# Supplementary figures and images for: Primary Cilium Depletion Typifies Cutaneous Melanoma In Situ and Malignant Melanoma
Source: PLoS One. 2011 Nov 11;6(11):e27410. doi: 10.1371/journal.pone.0027410 (PMC3214062; doi:10.1371/journal.pone.0027410)

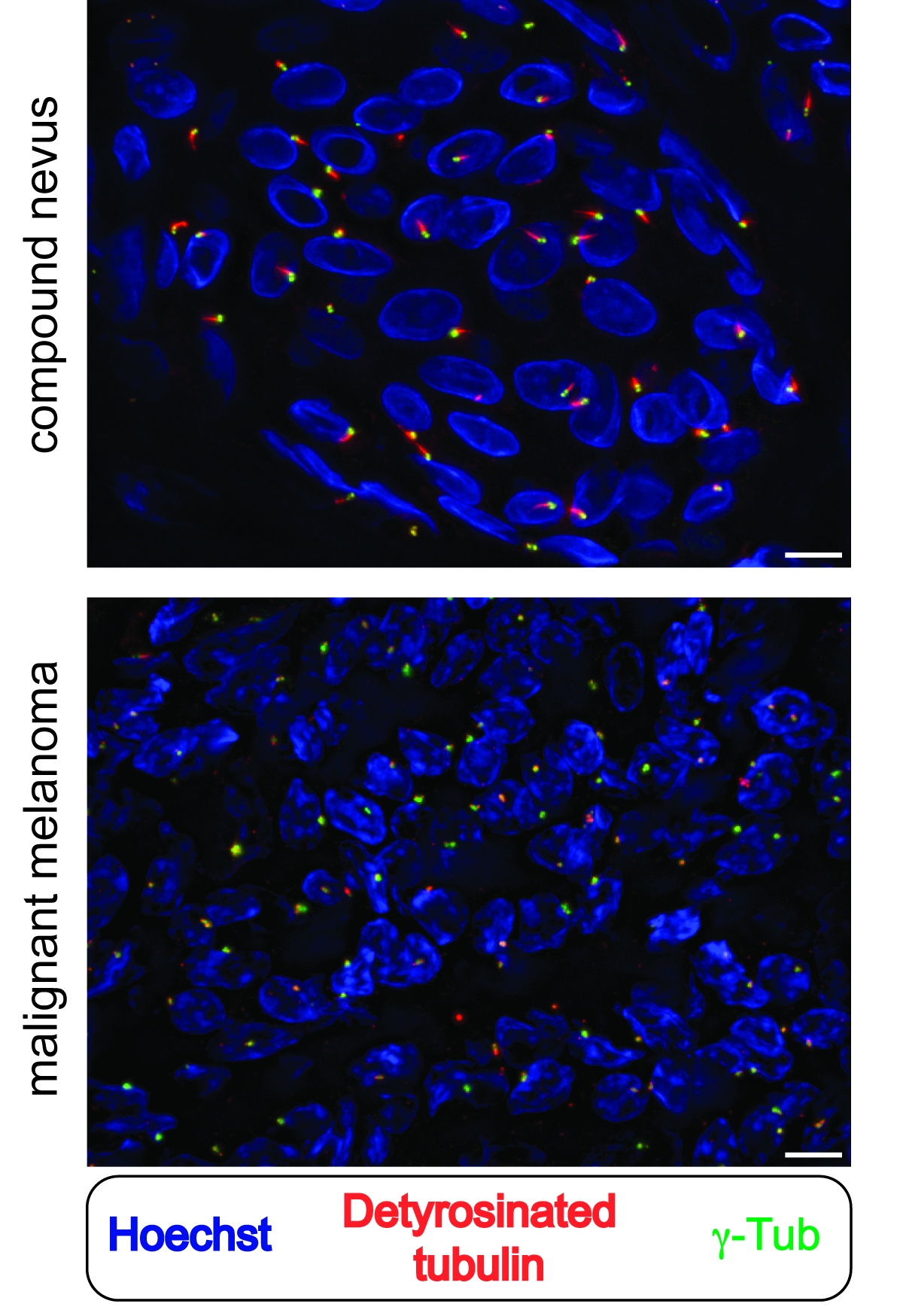

Supplement: Figure S1 — Detyrosinated tubulin antibodies show the same pattern of cilium loss in neoplastic melanocytes as do acetylated tubulin antibodies. Immunostaining and lesion types as shown. Scale bars: 20 µm. (TIF) [file pone.0027410.s001.tif]

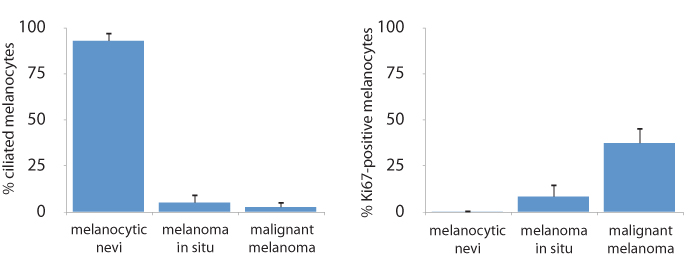

Supplement: Figure S2 — Proliferative indices do not fully account for the loss of the primary cilium amongst neoplastic melanocytes. (L) Average ciliation indices of melanocytic proliferations expressed as the percent of lesional melanocytes that are ciliated. (R) Average Ki67 proliferation indices of melanocytic proliferations expressed as the percent of lesional melanocytes that exhibit nuclear Ki67 immunoreactivity. Error bars: Standard deviation of mean. (JPG) [file pone.0027410.s002.jpg]
